# THE LTSS STATE SCORECARD AND AGE-FRIENDLY CARE

**DOI:** 10.1093/geroni/igad104.0589

**Published:** 2023-12-21

**Authors:** Rani Snyder

**Affiliations:** The John A. Hartford Foundation, New York City, New York, United States

## Abstract

Age-friendly care is health care that addresses a person’s unique needs and wants across the lifespan. It is a critical part of the age-friendly ecosystem. Rooted in the essential elements known as the “4Ms” (what Matters, Medication, Mentation, and Mobility), age friendly care is critical to successful delivery systems of health care as well as long-term services and supports. For the first time in its history, the LTSS State Scorecard will measure states as it relates to the presence of Age-Friendly Health System sites. Age-Friendly Health Systems is an initiative of The John A. Hartford Foundation and the Institute for Healthcare Improvement, and helps hospitals, doctor’s offices, retail pharmacy clinics, nursing homes, and home-care providers deliver age-friendly care. This portion of the symposium will describe Scorecard findings on Age-Friendly Health Systems and address more broadly the important role of age-friendly care in LTSS.

